# Predictors of Successful Testicular Sperm Extraction: A New Era for Men with Non-Obstructive Azoospermia

**DOI:** 10.3390/biomedicines12122679

**Published:** 2024-11-25

**Authors:** Aris Kaltsas, Sofoklis Stavros, Zisis Kratiras, Athanasios Zikopoulos, Nikolaos Machairiotis, Anastasios Potiris, Fotios Dimitriadis, Nikolaos Sofikitis, Michael Chrisofos, Athanasios Zachariou

**Affiliations:** 1 Third Department of Urology, Attikon University Hospital, School of Medicine, National and Kapodistrian University of Athens, 12462 Athens, Greece; ares-kaltsas@hotmail.com (A.K.); zkratiras@gmail.com (Z.K.); mxchris@yahoo.com (M.C.); 2 Third Department of Obstetrics and Gynecology, Attikon University Hospital, School of Medicine, National and Kapodistrian University of Athens, 12462 Athens, Greece; sfstavrou@yahoo.com (S.S.); nikolaosmachairiotis@gmail.com (N.M.); apotiris@med.uoa.gr (A.P.); 3 Department of Obstetrics and Gynecology, Royal Cornwall Hospital, Truro TR1 3LJ, UK; athanasios.zikopoulos1@nhs.net; 4 Department of Urology, Faculty of Medicine, School of Health Sciences, Aristotle University of Thessaloniki, 54124 Thessaloniki, Greece; helabio@yahoo.gr; 5 Laboratory of Spermatology, Department of Urology, Faculty of Medicine, School of Health Sciences, University of Ioannina, 45110 Ioannina, Greece; nsofikit@uoi.gr

**Keywords:** non-obstructive azoospermia, testicular sperm extraction, sperm retrieval predictors, male infertility, biomarkers, anti-Müllerian hormone, microRNAs, molecular diagnostics, seminal plasma, artificial intelligence

## Abstract

**Background/Objectives**: Non-obstructive azoospermia (NOA) is a severe form of male infertility characterized by the absence of sperm in the ejaculate due to impaired spermatogenesis. Testicular sperm extraction (TESE) combined with intracytoplasmic sperm injection is the primary treatment, but success rates are unpredictable, causing significant emotional and financial burdens. Traditional clinical and hormonal predictors have shown inconsistent reliability. This review aims to evaluate current and emerging non-invasive preoperative predictors of successful sperm retrieval in men with NOA, highlighting promising biomarkers and their potential clinical applications. **Methods**: A comprehensive literature review was conducted, examining studies on clinical and hormonal factors, imaging techniques, molecular biology biomarkers, and genetic testing related to TESE outcomes in NOA patients. The potential role of artificial intelligence and machine learning in enhancing predictive models was also explored. **Results**: Traditional predictors such as patient age, body mass index, infertility duration, testicular volume, and serum hormone levels (follicle-stimulating hormone, luteinizing hormone, inhibin B) have limited predictive value for TESE success. Emerging non-invasive biomarkers—including anti-Müllerian hormone levels, inhibin B to anti-Müllerian hormone ratio, specific microRNAs, long non-coding RNAs, circular RNAs, and germ-cell-specific proteins like TEX101—show promise in predicting successful sperm retrieval. Advanced imaging techniques like high-frequency ultrasound and functional magnetic resonance imaging offer potential but require further validation. Integrating molecular biomarkers with artificial intelligence and machine learning algorithms may enhance predictive accuracy. **Conclusions**: Predicting TESE outcomes in men with NOA remains challenging using conventional clinical and hormonal parameters. Emerging non-invasive biomarkers offer significant potential to improve predictive models but require validation through large-scale studies. Incorporating artificial intelligence and machine learning could further refine predictive accuracy, aiding clinical decision-making and improving patient counseling and treatment strategies in NOA.

## 1. Introduction

Non-obstructive azoospermia (NOA) is one of the most severe forms of male infertility, characterized by the absence of sperm in the ejaculate due to impaired spermatogenesis. Affecting approximately 1% of the male population and up to 10–15% of infertile men, NOA presents significant challenges for both diagnosis and treatment [1]. For couples desiring biological offspring, this condition poses a significant challenge, as natural conception is impossible. In such cases, testicular sperm extraction (TESE), particularly microdissection TESE (mTESE), is employed to retrieve sperm directly from the testes for use in assisted reproductive techniques such as intracytoplasmic sperm injection (ICSI) [2]. However, TESE is not always successful, with about 50% of patients failing to yield viable sperm despite undergoing invasive surgical procedures [3,4].

The unpredictability of sperm retrieval has important clinical implications, as TESE can result in emotional and financial burdens for patients and their partners [5]. While traditional clinical parameters—such as patient age, body mass index (BMI), duration of infertility, and testicular volume—along with hormonal assessments including follicle-stimulating hormone (FSH), luteinizing hormone (LH), and inhibin B levels are used to predict sperm retrieval success, their reliability is inconsistent. This is further complicated by the diverse causes of NOA, which hampers the development of universal predictive models [6,7].

Recent advances in molecular biology have introduced non-invasive biomarkers with potential for more accurate prediction. Biomarkers such as anti-Müllerian hormone (AMH) levels, the ratio of inhibin B to AMH (INHB/AMH), specific microRNAs (miRNAs), long non-coding RNAs (lncRNAs), circular RNAs (circRNAs), and germ-cell-specific proteins like TEX101 have shown promise in preliminary studies. These biomarkers, derived from serum or seminal plasma, offer the potential for more precise assessment of spermatogenic activity without the need for invasive procedures [8,9].

Additionally, advances in cellular, extracellular, and genetic testing methodologies have opened new avenues for assessing testicular function and the probability of sperm retrieval during TESE [10]. Artificial intelligence (AI) and machine learning (ML) algorithms are being integrated into these approaches to combine clinical and molecular data, offering further improvements in predictive accuracy and guiding clinical decisions [11,12]. Developing more reliable, non-invasive predictors is critical to reducing unnecessary surgical interventions and enhancing patient outcomes.

In this manuscript, we provide a comprehensive review of current and emerging preoperative predictors of successful sperm retrieval in men with NOA. We critically examine absolute clinical and hormonal predictive factors, molecular biology biomarkers, and cellular, extracellular, and genetic testing approaches. By synthesizing findings from recent research, we aim to highlight the most promising non-invasive biomarkers and discuss their potential application in clinical practice. Our goal is to synthesize these findings to aid clinicians in improving patient counseling, optimizing surgical decision-making, and ultimately enhancing reproductive outcomes.

## 2. Pre-Operative Predictors of TESE

The ability to predict the outcome of TESE in men with NOA is crucial for patient counseling and clinical decision-making. Accurate preoperative predictors can help identify patients who are more likely to benefit from TESE, thus avoiding unnecessary surgical interventions and associated risks. Over the years, various potential predictors have been explored, including clinical parameters, hormonal profiles, imaging techniques, molecular biomarkers, and genetic factors [13]. In this section, we comprehensively review the current evidence on pre-operative predictors of TESE success in NOA patients.

### 2.1. Absolute Clinical or Hormonal Predictive Factor

Several clinical and hormonal parameters have been studied to assess their potential predictive value for successful sperm retrieval in men undergoing TESE. These parameters include age, obesity and BMI, etiology of infertility, duration of infertility, and serum hormonal levels FSH, LH total testosterone (tT), prolactin (PRL), and inhibin B [14,15,16,17,18,19,20,21,22,23,24,25].

Age has been studied as a potential predictive factor for the sperm retrieval rate (SRR) in men undergoing TESE. While some studies have suggested that younger age may be associated with a higher likelihood of SRR [21], the results across broader populations have been mixed, with some studies showing no clear association between age and TESE outcomes [26,27]. However, in patients with Klinefelter syndrome (KS), younger age is consistently identified as a significant predictor of positive SRR outcomes during mTESE. This may be due to the preservation of spermatogenic foci in younger individuals, as spermatogenesis diminishes with age, especially in KS patients due to progressive testicular degeneration over time [28].

Obesity and BMI have also been examined as potential predictive factors influencing TESE success. While some studies have indicated a negative impact of obesity on in vitro fertilization (IVF) outcomes [29], the effect of male obesity on TESE outcomes remains inconclusive [30,31]. Higher BMI in men with NOA does not appear to significantly affect the SRR, although it may have an adverse effect on clinical pregnancy rates and early embryo development. However, these effects do not seem to result in significant differences in live birth rates or overall reproductive success. Therefore, while BMI may have some relevance, it is not a definitive predictor of TESE success or reproductive outcomes [23,32,33,34].

The etiology of NOA, including conditions such as KS, cryptorchidism, and varicocele, has been explored as a potential prognostic factor for SRR. Some studies suggest that patients with a known etiology of NOA (non-idiopathic), such as cryptorchidism or a history of mumps orchitis, may have higher SRR rates compared with those with idiopathic NOA [17,24]. KS, a chromosomal anomaly frequently found in men with azoospermia, is linked to lower SRR rates due to impaired spermatogenesis, although sperm retrieval is still possible with microsurgical techniques [35]. Furthermore, Y chromosome microdeletions, often present in azoospermic men, are associated with a decreased likelihood of SRR [36].

Cryptorchidism, particularly when untreated, negatively impacts SRR due to long-term damage to testicular tissue. Early surgical intervention, such as orchidopexy, can improve outcomes. Studies have shown that men with mild or unilateral forms of cryptorchidism, as well as those who undergo orchidopexy early in life, experience better SRR rates [37]. In contrast, severe or bilateral cryptorchidism and high-positioned testes often result in poorer SRR outcomes. Varicocele, another common cause of male infertility, is known to impair spermatogenesis and has a similarly negative effect on SRR, although surgical correction can improve results in some cases [38]. However, the predictive value of etiology alone is limited, and other factors need to be considered [39].

Testicular volume as a predictor of SRR has shown inconsistent results across different studies [21,40,41]. In men with NOA undergoing mTESE, several studies have found its predictive value to be limited [5,42,43]. However, in certain populations, such as transgender adolescents undergoing testicular biopsy, higher testicular volume (≥10 mL) has been associated with higher rates of sperm retrieval [44]. These findings suggest that testicular volume may hold more relevance in specific clinical contexts, though its overall predictive utility remains uncertain.

The duration of infertility has also been investigated as a potential predictive factor for SRR, with some studies suggesting that longer infertility durations may be associated with lower rates of SRR [15]. However, other research has not found a significant association between infertility duration and TESE outcomes [45]. This inconsistency indicates that the relationship between the length of infertility and SRR is not well established and may not be a reliable predictive factor, requiring further investigation to clarify its role in TESE success.

Serum hormonal levels, including FSH, LH, tT, PRL, and inhibin B, have been examined as potential predictive factors for TESE success. Some studies suggest that higher serum tT levels could be linked with higher SRR rates [46,47], while higher serum FSH levels, a hormone that stimulates Sertoli cellular secretory function and subsequently sperm production, may be associated with lower SRR rates [48]. A previous meta-analysis reported that serum FSH levels were a significant predictor of SRR success [43]. Conversely, a recent meta-analysis indicated that serum FSH levels did not significantly predict SRR success [41]. Other hormones, including LH, inhibin B, and estradiol, have also been assessed, but the findings were inconsistent [2,49]. Therefore, while hormonal imbalances can be indicative of underlying testicular dysfunction, their predictive value for TESE outcomes is limited [15,16].

While earlier studies focused on the predictive value of hormones like FSH, LH, and inhibin B, findings have often been conflicting. Recent research has shifted focus toward anti-Müllerian hormone (AMH) and its ratio to testosterone (AMH/tT) as more reliable predictors [50,51]. Notably, an AMH level below 4.62 ng/mL and an AMH/tT ratio lower than 1.02 have shown higher accuracy in predicting SRR [52].

Moreover, the ratio of inhibin B to AMH (INHB/AMH) has emerged as a promising non-invasive predictor of positive sperm retrieval in idiopathic NOA patients undergoing mTESE. In a study involving 168 men with idiopathic NOA, both INHB and the INHB/AMH ratio were identified as independent predictors of successful sperm retrieval, with the INHB/AMH ratio demonstrating superior predictive value [53]. Decision curve analysis indicated that utilizing the INHB/AMH ratio prior to mTESE enhanced the net benefit of positive sperm retrieval, surpassing the predictive utility of INHB alone. These findings suggest that combining inhibin B and AMH measurements may provide a more accurate hormonal predictor for SRR in idiopathic NOA patients [53].

Furthermore, serum 17α-hydroxyprogesterone (17αOH-P), a marker of steroidogenic function, has emerged as another promising predictor, particularly in patients undergoing FSH therapy. Studies suggest that lower pre-treatment levels of 17αOH-P (below 1.18 ng/mL) are associated with improved sperm parameters post-FSH administration, enhancing the chances of successful spermatogenesis in NOA patients [54].

In summary, no single clinical or hormonal factor currently provides a reliable prediction of TESE outcomes in men with NOA. Traditional predictors—including age, BMI, etiology of infertility, duration of infertility, and serum hormone levels like FSH, LH, and inhibin B—have demonstrated inconsistent or limited predictive value. While newer markers, such as anti-Müllerian hormone levels, the AMH/testosterone ratio, and the INHB/AMH ratio show potential, their efficacy as predictors still needs further validation. Given the limited predictive value of traditional clinical and hormonal factors, alternative approaches, such as imaging techniques, are being explored to enhance the prediction of TESE outcomes in NOA patients.

### 2.2. Sonography and MRI Perspectives

Imaging modalities such as ultrasound (US) and magnetic resonance imaging (MRI) have been explored as potential non-invasive predictive tools for sperm retrieval success in patients with NOA. These imaging techniques aim to assess testicular structure and function, providing insights that may correlate with spermatogenic activity and the likelihood of successful TESE.

Scrotal ultrasound is commonly employed to evaluate testicular and adjacent structures, aiming to detect pathologies such as varicoceles, testicular tumors, and tubular ectasia of the rete testis that may contribute to male infertility [55]. However, its role in predicting SRR in NOA patients remains limited. Studies have shown that US-based assessments of the epididymal head diameter provide limited clinical insights for patients diagnosed with NOA, but these assessments do not serve as a reliable predictor for the efficacy of TESE in these individuals [56].

The effectiveness of color-coded duplex US in predicting SRR among patients with NOA has been a subject of investigation, although its predictive accuracy remains inconclusive [57]. Recently, Ohta et al. explored the potential of high-frequency ultrasound (HFUS) to examine seminiferous tubules through high-resolution B-mode images in azoospermic patients [58]. Their comparison of HFUS images with corresponding histopathological findings from biopsy samples suggested that US images could offer stereoscopic insights due to their notably greater slice thickness. Notably, tubule diameters were frequently larger in diseased tissues when contrasted with US images in cases of obstructive azoospermia (AO) and Sertoli cell-only syndrome (SCOS), although this trend was not observed in other conditions. Such comparisons have shed light on SCOS predictability and revealed imaging characteristics, like inter-tubular gaps and reduced tubule diameters, indicative of testicular damage.

MRI, especially diffusion-weighted (DWI) and magnetization transfer MRI techniques, has proven useful in diagnosing male infertility and prognosticating TESE outcomes [59]. These functional MRI techniques enable the measurement of apparent diffusion coefficient (ADC) and magnetization transfer ratio, facilitating the evaluation of testicular hypospermatogenesis [59]. Proton magnetic resonance spectroscopy has also been investigated as a non-invasive way to predict spermatogenesis in males diagnosed with NOA [60]. One study observed significantly higher concentrations of phosphocholine in normal testes compared with those with SCOS [61]. Disparities were observed between patients with NOA and the general population when comparing ADC and fractional anisotropy (FA) levels in their testes, suggesting that these parameters may serve as valuable markers to detect severe impairments in spermatogenesis; however, no correlation has been observed between diffusion tensor imaging parameters and SRR in TESE procedures [62].

Therefore, while imaging tests, such as US and MRI, hold promise as potential predictive factors for SRR, further research is needed to validate and refine these techniques. Factors such as testicular volume, vascularization, ADC values, and metabolite concentrations measured by spectroscopy have shown potential, but more studies are required to establish their reliability and accuracy in predicting TESE outcomes in NOA patients [63,64].

In conclusion, imaging tests, such as US and MRI, have been examined as potential predictive indicators for SRR in NOA patients. While certain parameters like testicular volume and ADC values have shown some promise, further research must be completed in order to validate and refine them before being widely implemented in clinical practice.

### 2.3. Molecular Biology Biomarkers

Molecular biology parameters have also been explored as potential predictors for SRR in men with NOA. One such parameter is the sensitive, quantitative telomerase assay (SQTA), which measures telomerase activity within the sperm. This assay can track telomerase activity across different stages of embryo and somatic tissue development and differentiation. Typically, it increases during embryonic cell division and decreases during the differentiation of somatic tissues [65]. Telomerase plays a crucial role in preserving chromosomal stability by elongating telomeres. Studies show SQTA has high diagnostic accuracy in predicting the presence of testicular spermatozoa, with rates ranging from 84.2% to 90.2% in NOA men with varicoceles and 91.6% in those with non-mosaic Klinefelter syndrome [65,66]. These results suggest that the SQTA, with the appropriate threshold value, may have considerable predictive value in determining the SRR in men with NOA. However, further research is needed to confirm these findings.

The expression levels of germ-cell-specific mRNAs such as protamine 1 (PRM1), protamine 2 (PRM2), deleted in azoospermia (DAZ), and A-kinase anchoring protein 4 (AKAP4) have also been associated with SRR outcomes, with detectable levels linked to higher SRR rates in some studies [67]. Hashemi et al. identified that PRM1 showed an area under the curve (AUC) of 0.89, with a cut-off point of 0.39, providing a sensitivity of 87% and specificity of 90% in predicting successful sperm retrieval [67]. However, conflicting results exist, with other research finding no significant association [68].

Another promising molecular biomarker is TEX101, a germ-cell-specific protein found in semen. Jarvi et al. demonstrated that TEX101 concentrations below 0.2 ng/mL predicted a 0% chance of successful sperm retrieval in men with NOA, while levels above this threshold correlated with a 50% success rate (CI 34–66%; *p* < 0.05). This study identified TEX101 as the strongest non-invasive biomarker for predicting sperm retrieval success in NOA, offering high sensitivity and specificity [69].

Seminal haploid cell detection using flow cytometry has been shown to be a promising non-invasive predictive tool for TESE outcomes. Koscinski et al. demonstrated that flow cytometry achieved a sensitivity of 100% compared with 59% for cytology, indicating a high ability to detect cases where spermatogenesis is present [70]. However, flow cytometry’s specificity was lower, at 67%, as opposed to 83.5% for cytology, which may result in some false positives [70]. These findings suggest that while flow cytometry is highly sensitive, it is less specific than histology, which showed 100% specificity but only 50% sensitivity. Therefore, flow cytometry provides a sensitive, non-invasive alternative to predict spermatogenesis in men with NOA, potentially reducing the need for invasive biopsies.

Additional molecular biomarkers, such as extracellular vesicles (EVs) and microRNAs (miRNAs), have been explored as potential predictors for SRR [71]. Certain miRNAs (miR-34b/c, miR-449, and miR-122), which are related to different stages of sperm development, have been associated with SRR in some studies [72,73]. A study developed a predictive model using circulating microRNAs (hsa-miR-34b-3p, hsa-miR-34c-3p, hsa-miR-3065-3p, hsa-miR-4446-3p) that could effectively predict sperm retrieval success in NOA patients undergoing mTESE, with a high predictive accuracy (AUC = 0.927) [74]. This model offers a non-invasive alternative to current methods, potentially guiding clinical decision-making in NOA patients.

Long non-coding RNAs (lncRNAs) and circular RNAs (circRNAs) have also emerged as potential biomarkers. Cao et al. identified the CCDC37.DT-LOCI00505685 pair, with a sensitivity of 75% and specificity of 81.3% at a cut-off value of 0.677 (F1 = 0.78), and LOC440934-LOCI01929088, with 70.3% sensitivity and 93.8% specificity at a cut-off of 0.783 (F1 = 0.804). These findings suggest these exLncRNA pairs are promising biomarkers for SRR prediction, aiding in non-invasive assessment for NOA patients [75]. Similarly, the circular RNA circ_MGLL has been found to be inversely associated with successful sperm retrieval outcomes in idiopathic NOA patients, and a nomogram incorporating circ_MGLL expression, pathological type, and hormone levels demonstrated strong predictive performance (AUC = 0.857) [76].

Proteomic studies have highlighted lectin galactoside-binding soluble 3 binding protein (LGALS3BP) and other proteins as potential biomarker candidates [77]. According to findings by Fietz et al., specific proteins in the testes, when analyzed within seminal plasma, could serve as indicators of sperm presence and may help predict the SRR [78]. In SCOS patients with NOA, the expression levels of 42 seminal plasma proteins in seminal plasma were notably altered compared with healthy controls. Specifically, 28 proteins showed reduced expression levels, while 14 exhibited increased expression. Detailed tissue and cellular analyses pointed to testis-specific proteins such as LDHC, PGK2, DPEP3, and heat-shock proteins enriched in germ cells, including HSPA2 and HSPA4L, as promising markers of spermatogenic function. Among these, ZPBP2 and PGK2 expression levels have demonstrated reliability in predicting successful sperm retrieval and assessing sperm quality in NOA patients undergoing mTESE [79]. Additionally, proteins like LGALS3BP have shown promise as non-invasive biomarkers, with increased levels in seminal plasma linked to successful sperm retrieval, though further confirmation is needed [77].

Moreover, metabolomic profiling of seminal plasma has emerged as a novel approach to identifying non-invasive biomarkers for predicting SRR in NOA patients. Studies have identified distinct metabolomic fingerprints associated with positive and negative sperm retrieval outcomes, suggesting that metabolomics could provide valuable insights into spermatogenesis status [80,81]. However, these findings are preliminary, and larger studies are required to confirm the utility of metabolomics in this context.

Additionally, the ESX1 gene has been identified as a potential molecular marker for predicting sperm retrieval success in azoospermic men, especially those with NOA [82,83]. In a study of 81 azoospermic men, Bonaparte et al. found that ESX1 mRNA expression was present in 100% of patients with obstructive azoospermia, hypospermatogenesis, and incomplete maturation arrest, each of whom successfully had sperm retrieved. For cases of incomplete SCOS and complete maturation arrest, ESX1 was detected in 83% (5/6) and 67% (4/6) of cases, respectively, with sperm retrieval rates of 67% and 33%. By contrast, ESX1 was identified in only 19% of patients (3/16) with complete SCOS, where the retrieval success rate was 31% [84]. In focal spermatogenesis cases, ESX1 expression aligned with successful retrieval in 74% of patients undergoing TESE, but this correlation dropped to 27% among those who underwent microTESE, likely due to sampling differences. Additionally, patients expressing ESX1 exhibited lower methylation levels in the gene’s promoter region CpG islands than those without ESX1 expression, suggesting an epigenetic component in its predictive capacity. This evidence supports ESX1 as a reliable biomarker for detecting residual spermatogenesis in NOA-men, potentially guiding clinical choices in TESE procedures.

Despite encouraging findings, the use of molecular markers as SRR predictors in patients with NOA is still in its early stages, and further research is mandatory. Their expression may be influenced by various factors, including histopathological patterns, the duration of infertility, and comorbidities. Thus, while promising, the use of molecular markers should be interpreted cautiously and considered in conjunction with other clinical and histopathological parameters.

In summary, several molecular biology parameters, including the sensitive, quantitative telomerase assay, gene expression ratios, proteins like TEX101, haploid cell detection via flow cytometry, proteomic biomarkers like LGALS3BP, miRNAs, lncRNAs, circRNAs, and the ESX1 gene and histone demethylase expression, have shown promise as predictive indicators for successful sperm retrieval in males diagnosed with NOA. Omics techniques applied to seminal plasma—encompassing genomics, transcriptomics, proteomics, and metabolomics—offer a comprehensive strategy for identifying non-invasive biomarkers to predict SRR outcomes. While mRNAs in seminal plasma have shown limited predictive value due to degradation issues, miRNAs and lncRNAs have demonstrated higher stability and potential as reliable biomarkers. Proteomic and metabolomic analyses have also identified candidate biomarkers, but further large-scale studies are needed to validate their clinical utility [85]. However, further research is needed to validate these parameters and to determine their practical utility in clinical settings.

### 2.4. Cellular, Extracellular, and Genetic Testing

Cellular, extracellular, and genetic testing have been explored as potential predictive factors for SRR in men with NOA. These tests aim to provide valuable information about the testicular function and the likelihood of finding spermatozoa during TESE procedures.

Cellular testing involves the examination of testicular tissue to assess the presence and quality of spermatogenic cells. Testicular histopathology, including the evaluation of histological patterns, has been investigated as a predictive factor for SRR [86,87]. Studies have shown that the presence of haploid cells, such as spermatozoa or spermatids, in diagnostic biopsies has been found to be predictive of SRR in subsequent testicular biopsies [87,88]. For instance, patients with hypospermatogenesis or maturation arrest have been found to have higher SRR rates compared with those with SCOS or tubular sclerosis [87,89]. Despite these findings, the application of testis histology still has limitations. It can only be assessed post surgery, which restricts its use in pre-surgical counseling regarding SRR probability. Additionally, it may not accurately represent the entire testicular tissue due to the potential variance in histopathological patterns within the same testis [90]. Nevertheless, in certain cases where a diagnostic testicular biopsy has been previously performed, testis histology could provide useful insights for patient counseling and prediction of SRR success [91]. However, its post-surgical acquisition and potentially unrepresentative nature limit its utility.

Seminal plasma functions as a liquid biopsy for the male reproductive system, comprising secretions from various sources, including the testes, epididymides, seminal vesicles, prostate, and bulbourethral glands. It contains a complex mixture of proteins, metabolites, cell-free nucleic acids, and microvesicles, all intricately linked to gonadal activity. Although numerous studies have investigated potential biomarkers within seminal fluid, their routine use in clinical settings remains limited. This limitation may stem from the complex interaction between clinical and genetic factors associated with NOA, which likely complicates the identification of definitive markers for residual spermatogenesis [78]. Integrating clinical data with biomarker information could, therefore, improve the ability to predict surgical outcomes and assist in decision-making for NOA patients. 

Extracellular testing encompasses the examination of biomarkers found within seminal plasma or extracellular vesicles. In a study conducted by Han et al., a specific type of extracellular vesicle was discovered originating from seminal plasma, known as tRNA-derived small RNA (tsRNA) [92]. This tsRNA may potentially be used as a biomarker for the SRR in males with NOA [92]. These extracellular vesicles were found to have differential expression patterns between men with SRR and those with failed sperm retrieval. Moreover, cell-free nucleic acids (cfDNA and cfRNA) in seminal plasma have been explored as potential non-invasive biomarkers for spermatogenesis status. Elevated levels of cfDNA in seminal plasma are linked to sperm abnormalities, suggesting their potential use in predicting SRR outcomes. However, further validation and standardization are required to make these biomarkers reliable for clinical use [93,94,95].

The use of genetic testing has been investigated as a potential prognostic indicator for SRR in males diagnosed with NOA. Numerous studies have explored the significance of genetic markers, specifically Y chromosome microdeletions and gene expression ratios, in predicting the presence of spermatozoa during TESE. Approximately 8–12% of NOA cases are related to Y chromosome microdeletions, particularly in the azoospermia factor (AZF) regions [96,97,98]. These deletions are classified into three regions, AZFa, AZFb, and AZFc, each associated with distinct spermatogenic outcomes [99,100]. The whole deletion of the AZFa region correlates with a severe testicular phenotype known as SCOS, while full deletions of the AZFb area are linked to maturation arrest. Complete deletions encompassing both the AZFa and AZFb regions are associated with a poor prognosis for sperm retrieval with TESE; consequently, TESE should be avoided in these individuals [101,102]. In contrast, deletions in the AZFc region result in a phenotypic spectrum that ranges from azoospermia to oligozoospermia. Testicular sperm is present in 50–75% of males with AZFc microdeletions [101,102,103]. Men with AZFc microdeletions who are oligo-azoospermic or have sperm retrieved after TESE must be advised that any male progeny will inherit the deletion [104].

Genetic profiling through whole-exome sequencing (WES) has improved the diagnosis of genetic contributors to NOA [105]. The expression of specific gene transcripts, such as CDY1 and BOULE, in testicular tissue has been evaluated as potential biomarkers for predicting sperm retrieval success. A study found that assessing both CDY1 and BOULE together improved predictive specificity for sperm presence during TESE, suggesting that using these two markers could enhance the success rates of sperm retrieval [106].

In summary, cellular, extracellular, and genetic testing have shown promise as potential predictive factors for successful sperm retrieval in men with NOA. These tests provide valuable information about testicular function, histopathology, biomarker expression, and genetic factors that may influence the success of TESE procedures. However, further research is needed to validate and refine these tests and to determine their utility in clinical practice.

To further clarify the potential value and limitations of these various predictors, Table 1 compares traditional clinical and hormonal factors with emerging molecular biomarkers and imaging techniques in the context of TESE success prediction in NOA patients.

## 3. Artificial Intelligence and Machine Learning in NOA Diagnostics and Treatment

**Figure 1:**
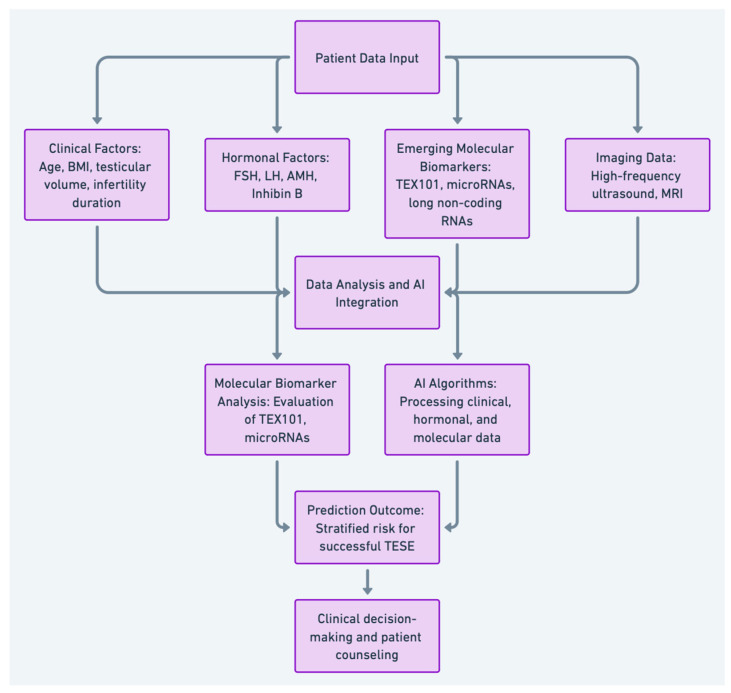
Flowchart of TESE success prediction in NOA patients using clinical data, molecular biomarkers, imaging techniques, and AI.

The integration of AI and ML into clinical and genetic data analysis offers a transformative approach to identifying non-invasive biomarkers for predicting sperm retrieval in NOA. AI techniques, such as machine learning, have shown significant promise in improving diagnostic accuracy and personalized treatment plans by analyzing vast amounts of clinical, genomic, proteomic, and metabolomic data, potentially leading to better reproductive outcomes [107]. Figure 1 provides a flowchart that illustrates the process of predicting TESE success in NOA patients. It demonstrates how clinical parameters, molecular biomarkers, imaging techniques, and AI are integrated to enhance predictive accuracy, guiding clinical decision-making and improving patient outcomes.

Several studies highlight the potential of AI in this domain. For example, the use of machine learning models to predict successful sperm retrieval in NOA patients has demonstrated remarkable performance. Bachelot et al. implemented eight machine learning models to predict sperm retrieval outcomes from preoperative clinical data, identifying random forest as the best-performing model with an AUC of 0.90 and 100% sensitivity. Inhibin B levels and the presence of varicocele were found to be strong predictors [108].

AI has also been applied in real-time sperm identification during TESE procedures. In a recent study, Goss et al. demonstrated that AI-powered image analysis significantly reduced the time required to identify sperm in complex testicular tissue samples compared with manual identification by embryologists. The AI model improved sperm detection speed by over 1000-fold while maintaining high accuracy [93].

Moreover, Tang et al. utilized multiple machine learning techniques, including XGBoost, to analyze NOA datasets and identify signature genes such as IL20RB and DZIP1, which were strongly linked to NOA. The study integrated microarray data with AI-driven differential gene analysis, providing deeper insights into the genetic underpinnings of NOA [109].

While AI holds great promise, challenges remain in its implementation. One major issue is the need for extensive, high-quality datasets to avoid model overfitting and enhance generalizability. Ensuring robust validation with external datasets across diverse demographics and clinical settings is critical for AI to achieve clinical utility in NOA diagnostics. Additionally, understanding the intricate interplay of genetic, hormonal, and environmental factors affecting spermatogenesis is vital for refining AI models.

In conclusion, the application of AI in NOA is poised to revolutionize diagnostic and therapeutic strategies, moving toward a more personalized and data-driven approach to male infertility management. However, continued advancements in data collection, model validation, and interdisciplinary collaboration are necessary to fully unlock AI’s potential in this field.

## 4. Challenges and Future of Non-Invasive Biomarkers in NOA

The integration of non-invasive biomarkers into the sperm retrieval process for NOA patients holds great potential for revolutionizing male infertility treatment. However, there are significant challenges and limitations that must be addressed to fully realize this potential.

The first major step involves comprehensive validation of the predictive value of these biomarkers across diverse populations. While initial studies have shown promise, large-scale, prospective clinical trials are necessary to confirm the correlation between biomarker levels and the presence, quality, and quantity of sperm in NOA patients. These studies must account for variations in age, ethnicity, and the underlying etiology of NOA, which may significantly impact biomarker reliability. Bachelot et al. demonstrated that machine learning models like random forest algorithms can predict sperm retrieval outcomes with high accuracy but emphasized the need for multicenter validation studies to establish broader clinical utility [108].

Furthermore, technological advancements are essential for the effective use of these biomarkers in clinical practice. The development of rapid, user-friendly diagnostic tools will be crucial for integrating biomarker assessments into routine medical workflows. These tools should enable quick and accurate evaluation of biomarker profiles, reducing delays in diagnosis and treatment decision-making. For instance, Zarezadeh et al. emphasized the importance of using omics technologies to analyze seminal plasma for non-invasive biomarkers, which could significantly streamline diagnostic procedures and lower the risk of unnecessary surgeries [110].

Collaboration among interdisciplinary teams will be vital to ensure the successful development and implementation of these technologies. A cooperative effort between reproductive endocrinologists, geneticists, molecular biologists, bioinformaticians, and imaging specialists will provide the comprehensive expertise necessary to create robust, accurate, and reliable biomarker-driven diagnostic tools.

Ethical and regulatory considerations also play a critical role in the future of biomarker integration. The collection and use of genetic and molecular data come with significant privacy concerns, particularly given the sensitive nature of reproductive health. Amer et al. highlight the importance of establishing strict ethical guidelines and protocols for the use of non-invasive biomarkers, ensuring that patient data is handled with care and transparency [111]. Additionally, consent processes must be transparent, ensuring that patients fully understand how their data will be used and the implications for their treatment.

While the potential benefits of AI and biomarker-driven diagnostics are significant, data quality and generalizability remain major challenges. Current AI models rely on relatively small and homogenous datasets, which may limit their applicability in broader clinical settings. For example, Tang et al. successfully utilized machine learning techniques to identify gene networks associated with NOA, but they caution that the generalizability of these findings is limited without more diverse datasets [109].

Another significant limitation involves the complexity of NOA etiology. NOA is a heterogeneous condition with diverse genetic, hormonal, and environmental causes. As a result, no single biomarker or set of biomarkers may be universally predictive. Further research is required to develop multifactorial models that incorporate not only molecular biomarkers but also clinical and hormonal data. These comprehensive models could enhance the accuracy of sperm retrieval predictions and provide more tailored treatment recommendations.

Lastly, the cost and accessibility of biomarker-driven diagnostics may limit their widespread adoption. Advanced molecular and genetic testing is often expensive and may not be readily available in all healthcare settings. Ensuring that these new technologies are affordable and accessible to a wide range of patients will be critical for their integration into standard care. This may require policy changes and funding initiatives to support the development and distribution of these technologies in both high-income and resource-limited settings.

In conclusion, the future of non-invasive biomarkers in NOA diagnostics and treatment is promising but requires significant advancements in clinical validation, technological development, and ethical oversight. Addressing these limitations through interdisciplinary collaboration and rigorous research will enable the transition to more personalized, accurate, and patient-centered care for men with NOA. Ultimately, integrating these biomarkers could significantly improve the outcomes of sperm retrieval procedures, reduce unnecessary surgeries, and enhance the overall efficacy of reproductive therapies.

## 5. Conclusions

In men with NOA, the success of TESE remains challenging to predict due to the lack of definitive preoperative predictors. Traditional clinical and hormonal factors have demonstrated inconsistent or limited predictive value. Recent advances in molecular biology have identified several promising non-invasive biomarkers, including TEX101, miRNAs, lncRNAs, circRNAs, and gene expression markers such as ESX1, CDY1, and BOULE, which have shown potential in predicting successful sperm retrieval. Omics techniques applied to seminal plasma—including genomics, transcriptomics, proteomics, and metabolomics—offer a comprehensive and non-invasive strategy for identifying biomarkers that predict TESE outcomes. While preliminary findings are encouraging, further large-scale, prospective studies are necessary to validate these biomarkers and integrate them into clinical practice. Imaging techniques and cellular, extracellular, and genetic testing also offer valuable insights but require further validation. The integration of AI and machine learning algorithms holds significant promise in combining clinical data with biomarkers to enhance predictive accuracy and guide clinical decision-making. Collaborative efforts and large-scale studies are essential to validate these biomarkers and incorporate them into clinical practice, ultimately improving patient counseling and treatment strategies in NOA.

## Figures and Tables

**Table 1:** Comparison of traditional and emerging predictors of TESE success in NOA patients.

Predictor Type	Specific Markers/Factors	Predictive Value	Limitations
Traditional Clinical Factors	Age, BMI, Infertility Duration, Testicular Volume	Limited predictive accuracy. Younger age may correlate with better outcomes, especially in Klinefelter syndrome patients.	Inconsistent results across populations; not reliable in isolation.
Hormonal Factors	FSH, LH, Testosterone, Inhibin B	Mixed results. FSH often linked with lower success rates, but inconsistency in findings.	Lack of universal reliability; varies widely between individuals.
Emerging Molecular Biomarkers	TEX101, AMH, microRNAs (e.g., miR-34b/c)	Promising in preliminary studies; could enhance non-invasive prediction.	Requires large-scale validation before clinical application.
Imaging Techniques	High-frequency ultrasound, MRI	Can provide structural insights, some evidence for correlation with spermatogenic activity.	Needs further research and refinement for clinical use.

## Data Availability

Not applicable.
